# Assessment of the Role of PAL in Lignin Accumulation in Wheat (*Tríticum aestívum* L.) at the Early Stage of Ontogenesis

**DOI:** 10.3390/ijms22189848

**Published:** 2021-09-12

**Authors:** Pavel Feduraev, Anastasiia Riabova, Liubov Skrypnik, Artem Pungin, Elina Tokupova, Pavel Maslennikov, Galina Chupakhina

**Affiliations:** Institute of Living Systems, Immanuel Kant Baltic Federal University, 236040 Kaliningrad, Russia; avryabova@stud.kantiana.ru (A.R.); lskrypnik@kantiana.ru (L.S.); apungin@kantiana.ru (A.P.); ishtrants@kantiana.ru (E.T.); pmaslennikov@kantiana.ru (P.M.); gchupakhina@kantiana.ru (G.C.)

**Keywords:** *Triticum aestivum* L., lignin, L-phenylalanine, L-tyrosine, D-phenylalanine, O-Benzylhydroxylamine, PAL, POD

## Abstract

The current study evaluates the role of phenylalanine ammonia-lyase (PAL) and the associated metabolic complex in the accumulation of lignin in common wheat plants (*Tríticum aestívum* L.) at the early stages of ontogenesis. The data analysis was performed using plant samples that had reached Phases 4 and 5 on the Feekes scale—these phases are characterized by a transition to the formation of axial (stem) structures in cereal plants. We have shown that the substrate stimulation of PAL with key substrates, such as L-phenylalanine and L-tyrosine, leads to a significant increase in lignin by an average of 20% in experimental plants compared to control plants. In addition, the presence of these compounds in the nutrient medium led to an increase in the number of gene transcripts associated with lignin synthesis (*PAL6*, *C4H1*, *4CL1*, *C3H1*). Inhibition was the main tool of the study. Potential competitive inhibitors of PAL were used: the optical isomer of L-phenylalanine—D-phenylalanine—and the hydroxylamine equivalent of phenylalanine—O-Benzylhydroxylamine. As a result, plants incubated on a medium supplemented with O-Benzylhydroxylamine were characterized by reduced PAL activity (almost one third). The lignin content of the cell wall in plants treated with O-Benzylhydroxylamine was almost halved. In contrast, D-phenylalanine did not lead to significant changes in the lignin-associated metabolic complex, and its effect was similar to that of specific substrates.

## 1. Introduction

Wheat (*Triticum aestivum* L.) is the most cultivated crop on earth [1,2]. However, monoculture remains the most common agricultural practice in its cultivation [3,4]. Monoculture is characterized by several fundamental disadvantages, including a decrease in resistance to environmental factors [5]. Thus, increasing wheat sustainability remains a vital problem of modern science-intensive agrobiotechnology.

One possible solution for this problem implies improving so called systemic acquired resistance (SAR) in plants. The SAR in plants—which can be considered a complex of metabolic and functional states—is manifested, inter alia, through the intensification of the biosynthesis of barrier structures of the cell wall, such as lignin. Lignin is a complex branched phenolic biopolymer that is mainly deposited in the secondary cell wall at later stages of cell differentiation. It displaces the aqueous phase of the cell wall, coating cellulose and matrix polysaccharides, provides increased mechanical strength and forms a waterproof barrier of the plant cell [6]. Lignin is also required to strengthen the cells of vascular tissues that carry water under negative pressure resulting from transpiration. The importance of lignin in these tissues is demonstrated by vascular collapse in lignin-deficient plants [7]. Lignin biosynthesis, and, consequently, the formation of additional barrier cell structures, is one of the key innovations in the evolution of vascular plants.

Along with the ability to provide mechanical support for axial structures and stabilize the plant’s vascular tissue system, the lignin structures of the cell wall act as a physical barrier preventing pathogens from entering the plant cell [8,9]. It was shown that the expression of genes for lignin synthesis increased in response to pathogen exposure [10]. Evaluation of plants in which genes for biosynthesis of monolignols were suppressed or over-expressed showed that lignin formation is important for disease resistance [11]. Thus, lignin not only determines plant resistance to biotic invasion, but also largely ensures normal plant growth and development [12].

Canonically, lignin is synthesized via the phenylalanine-mediated metabolic pathway. In this process, L-phenylalanine (L-Phe)—an aromatic proteinogenic amino acid—is used as the main substrate for lignin formation. Phenylalanine ammonia-lyase (PAL) converts it to cinnamate. Thus, PAL is the first regulator of phenylpropanoid metabolism [13]. However, an alternative “shortcut” pathway based on tyrosine ammonia-lyase has been discovered recently, which is more energy efficient. The inclusion of L-tyrosine (L-Tyr) in the processes of lignin formation is also carried out by PAL. It is important to note that the tyrosine-mediated pathway of lignin formation is characteristic only of cereals [14,15,16].

The substrate specificity of PAL is the basis for new methods to enhance the formation of lignin structures. For example, it was previously shown that treatment of plants with precursors of phenylpropanoid synthesis (phenylalanine and tyrosine) led to a significant increase in the number of phenols of various types, an increase in the phenylalanine ammonia-lyase activity, and an increase in the expression of lignin-associated genes [17].

One of the main problems in understanding the functional role of PAL in the formation of lignin structures of wheat is the complexity and specificity of the involvement of various genes of the *PAL* gene family in the process of lignin formation during different stages of plant development. However, inhibition of the enzymatic products of these genes allows us to solve this issue [18]. Hydroxylamine equivalents of phenylalanine are widely used for this purpose (for example, O-Benzylhydroxylamine (OBHA) [19]). In addition, Bazukyan et al. showed that D-phenylalanine (D-Phe), the optical isomer of L-phenylalanine, can also act as a potential inhibitor of PAL [20].

Thus, the use of specific competitive inhibitors, in comparison with substrates, should make it possible to assess the participation of PAL in the formation of lignin structures of the cell wall in the early stages of ontogenesis (during the period of laying stem structures). Moreover, this approach will allow one to partially evaluate metabolic changes associated with the process of lignin formation (peroxidase activity, hydrogen peroxide level, expression of lignin-associated genes) of cereal plants, using wheat (*Triticum aestivum* L.) as an example. We assume that the use of specific competitive inhibitors of PAL will lead to a decrease in the level of lignin in the cell wall, as well as affect the general metabolism of phenylpropanoids by means of altering the expression of lignin-associated genes. On the contrary, stimulation of PAL by typical substrates should lead to the intensification of lignin accumulation.

## 2. Results

### 2.1. Effect of Substrates and Potential Inhibitors on Plant Growth and Biomass of Wheat

The use of specific agents for the treatment of experimental wheat plants led to significant (*p* ≤ 0.05) changes in the growth and accumulation of biomass (Table 1).

Plant height and shoot weight were assessed when plants reached phase 4–5 according to Feekes scale. The seed germination parameter was evaluated 3–4 days after placing the seeds in the experimental solutions. It should be noted that the presence of both substrates and inhibitors did not have a significant (*p* ≤ 0.05) effect on seed germination in comparison with the control. However, changes become apparent by the time the plants reach the desired developmental stage. Thus, plants exposed to nutrient media containing substrate substances demonstrated significantly (*p* ≤ 0.05) more intensive growth and biomass gain. On the other hand, plants cultivated on a medium enriched with OBHA show limited growth, and the dry matter content is significantly lower in comparison with other variants of the experiment.

### 2.2. PAL and TAL Activities

**Figure 1:**
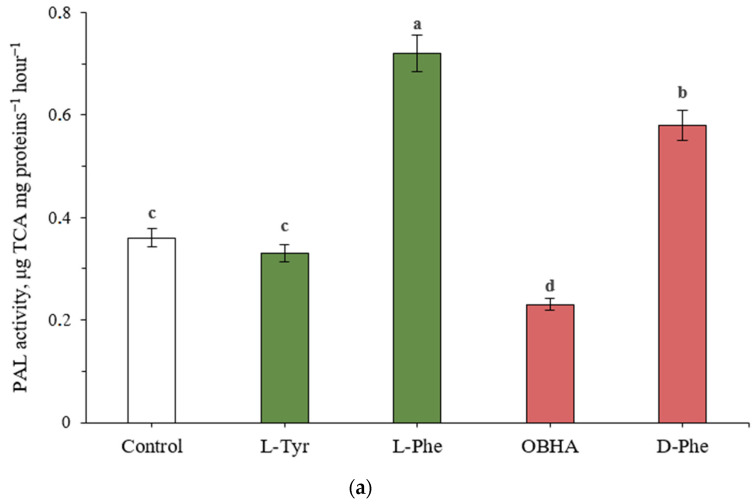

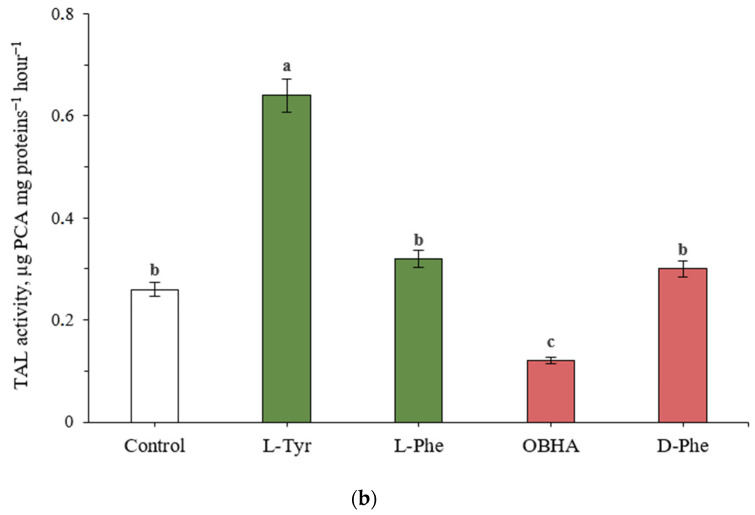
Activity of PAL (**a**) and TAL (**b**) in wheat samples incubated on a nutrient medium supplemented with 500 μM of active compounds. Bars marked with different letters show significant differences at (*p* ≤ 0.05) according to Tukey’s test.

Initially, the activity of the key enzymes in the biosynthesis of phenolic compounds was assessed (Figure 1). Since the PAL of cereal plants can use tyrosine as an alternative substrate, the tyrosine ammonia-lyase activity of plant extracts was also assessed.

A significant (*p* ≤ 0.05) increase in the PAL and TAL activities was observed in plants exposed to L-phenylalanine and L-tyrosine (the results were measured in the corresponding equivalent units: trans-cinnamic acid (TCA) for PAL and para-coumaric acid (PCA) for TAL). Experimental “Vanek” wheat samples, transferred to a nutrient medium containing 500 μM phenylalanine and tyrosine and exposed under such conditions during the entire period of growth, showed a significantly larger than two-fold increase in the activity of PAL and TAL, respectively. The rate of tyrosine involvement in the metabolism through the tyrosine ammonia-lyase activity of PAL was slightly lower (0.64 µg PCA mg proteins^−1^ h^−1^ in comparison with the level of control plants: 0.26 µg PCA mg proteins^−1^ h^−1^) than the rate of phenylalanine involvement (0.72 µg TCA mg proteins^−1^ h^−1^ in comparison with the level of control plants: 0.36 µg TCA mg proteins^−1^ h^−1^). Conversely, there was no significant increase in the PAL enzymatic activity in plants grown on a nutrient medium containing 500 μM L-tyrosine, nor an increase in TAL activity for plants incubated on a nutrient medium supplemented with L-phenylalanine.

The addition of the OBHA inhibitor to the nutrient medium resulted in a significant decrease in both phenylalanine- and tyrosine-ammonia-lyase activity of the extracts in comparison with the control group plants (0.23 TCA mg proteins^−1^ h^−1^ and 0.12 µg PCA mg proteins^−1^ h^−1^). On the other hand, the addition of D-Phe to the nutrient medium did not lead to decreased activity in both versions of the experiment. TAL activity in plants exposed to D-Phe did not significantly differ from the control group, while PAL activity significantly increased (0.58 µg TCA mg proteins^−1^ h^−1^ in comparison with the level of control plants: 0.36 µg TCA mg proteins^−1^ h^−1^).

### 2.3. POD Activity

There are two principal stages in the synthesis of lignin structures: biosynthesis of monolignol initiated by PAL, and polymerization of monolignol due to free radical coupling. The latter is characterized by the work of redox-active enzymes, particularly POD [21].

**Figure 2:**
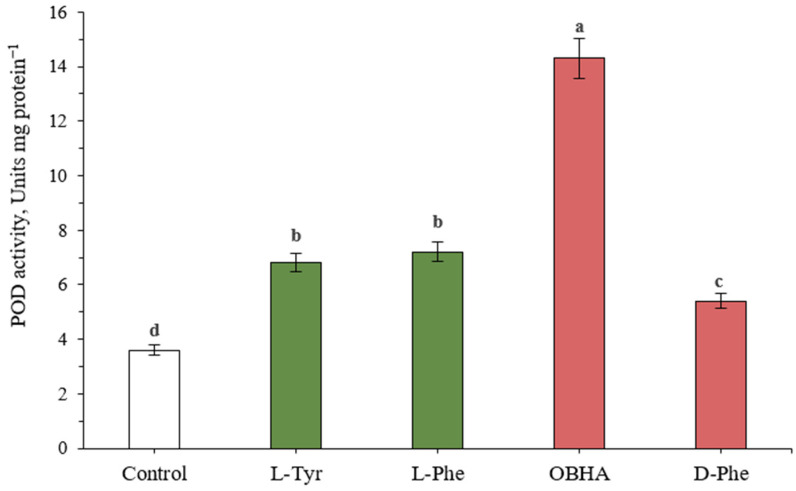
POD activity in wheat samples incubated on a nutrient medium supplemented with 500 μM of active compounds. Bars marked with different letters show the significant differences at (*p* ≤ 0.05) according to Tukey’s test.

It was shown that POD activity increased in the presence of both substrates (Figure 2). Plants grown on a phenylalanine-enriched medium were characterized by a larger than two-fold increase in POD activity (7.2 unit/mg protein in comparison with level of control plants: 3.5 unit/mg protein). A similar trend was observed when plants were incubated in the presence of tyrosine (6.8 unit/mg protein in comparison with level of control plants: 3.5 unit/mg protein).

However, the highest peroxidase activity was demonstrated by extracts of plants grown in the presence of the OBHA—the PAL inhibitor. A larger than 4-fold increase in POD activity has been noted (14.2 unit/mg protein in comparison with level of control plants: 3.5 unit/mg protein).

The selected methods made it possible to use the same extracts in the evaluation of the PAL/TAL activity as well as the POD activity. Growing plants under conditions of constant exposure to aromatic proteinogenic amino acids led to a significant (*p* ≤ 0.05) increase in the activity of the selected groups of enzymes. At the same time, the presence of D-Phe in the medium had a similar effect. However, it is important to remember that these enzymatic systems are involved in the metabolism of phenylpropanoids at different stages.

### 2.4. Hydrogen Peroxide Level

**Figure 3:**
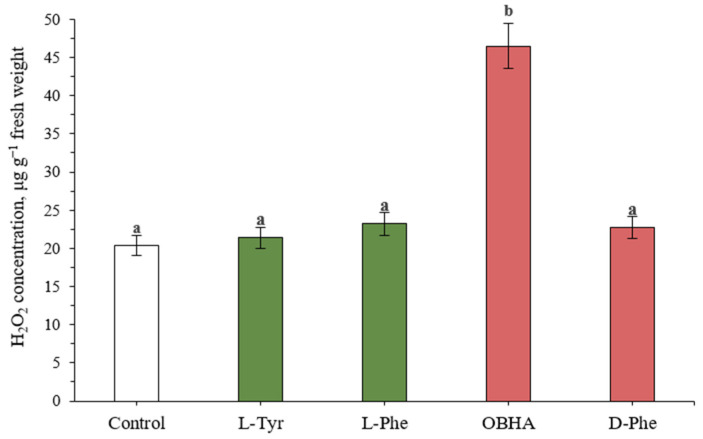
Concentration of hydrogen peroxide in wheat samples incubated on a nutrient medium supplemented with 500 μM of active compounds. Bars marked with different letters show the significant differences at (*p* ≤ 0.05) according to Tukey’s test.

One of the substrates of POD in lignin formation, along with monolignols, is hydrogen peroxide. It is assumed that an increase in peroxidase activity associated with the intensification of lignin formation should lead to increased production of hydrogen peroxide [22]. In practice, plants exposed to the substrate substances (L-Phe, L-Tyr) and to D-Phe do not show a significant increase in hydrogen peroxide levels compared to control (Figure 3).

On the other hand, the high peroxidase activity in plants grown on a nutrient medium with a hydroxylamine equivalent of phenylalanine correlates with a high level of hydrogen peroxide in the same plants (46.5 μg g^−1^ fresh weight in comparison with level of control plants: 20.4 μg g^−1^ fresh weight).

### 2.5. Lignin Content

**Figure 4:**
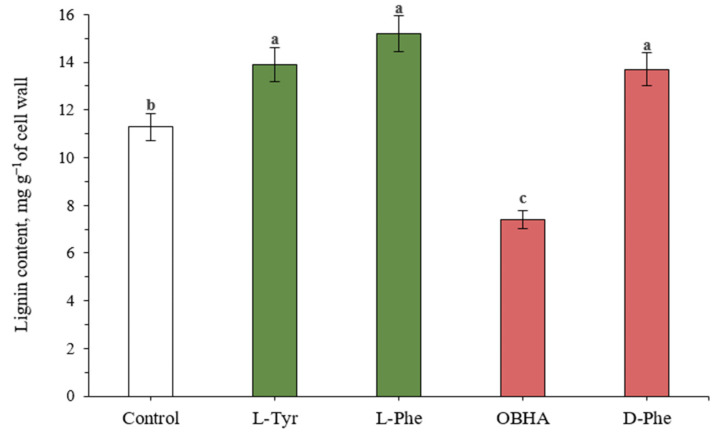
Lignin content in wheat samples incubated on a nutrient medium supplemented with 500 µM of active compounds. Bars marked with different letters show significant differences at (*p* ≤ 0.05) according to Tukey’s test.

An increase in the activity of key enzymes—the ones responsible for the synthesis of lignin structures (PAL) and subsequent condensation of lignin monomers (POD)—led to a significant increase in the lignin level in the cell walls of experimental plants exposed to L-Tyr, L-Phe, and D-Phe (Figure 4).

Plant samples incubated on nutrient media supplemented with the substrate amino acids—PAL effectors—demonstrate significantly (*p* ≤ 0.05) more efficient lignin accumulation at this stage of ontogenesis (on average by 20% compared to the control). The introduction of D-Phe into the solution also leads to a significant increase in the level of cell wall lignin (13.7 mg g^−1^ of cell wall in comparison with level of control plants: 11.3 mg g^−1^ of cell wall). Despite the high peroxidase activity (which was probably caused by a number of external factors), the lignin content of the cell walls in plants incubated on the OBHA-enriched medium was significantly lower than the control (7.4 mg g^−1^ of cell wall) in comparison with the level of control plants (11.3 mg g^−1^ of cell wall).

It should be noted that Phases 4 and 5 of plant development on the Feekes scale are characterized by an active formation of a secondary cell wall (determined by the presence of lignin in its structure). The intensification of this process can become an excellent foundation for the successful growth and development of a plant at later stages of ontogenesis.

### 2.6. Gene Expression

**Figure 5:**
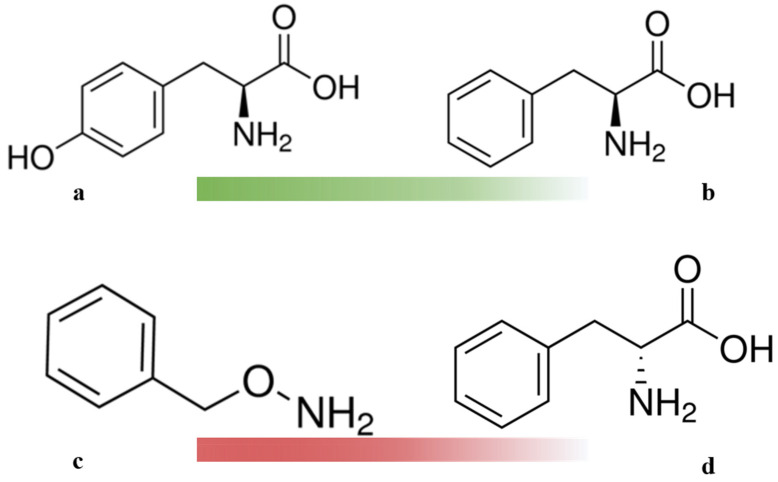
Active compounds used in the experiment: substrates L-Tyrosine (**a**) and L-Phenylalanine (**b**); inhibitors O-Benzylhydroxylamine (**c**) and D-Phenylalanine (**d**).

**Figure 6:**
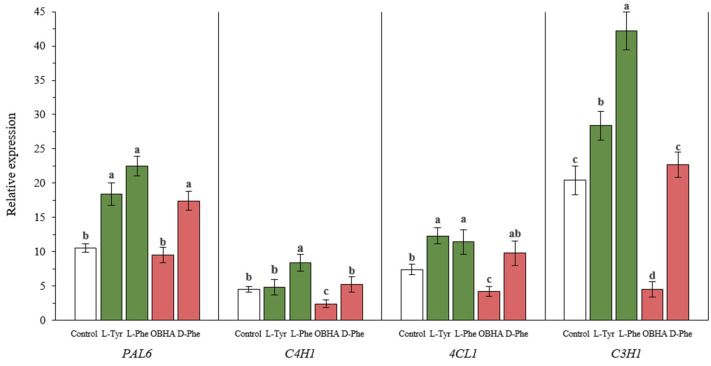
Relative quantitative values of *PAL6*, *C4H1*, *4CL1* and *C3H1* transcripts in wheat plants incubated on a nutrient medium supplemented with 500 μM of active compounds. Bars marked with different letters show significant differences at (*p* ≤ 0.05) according to Tukey’s test.

To better understand the role of aromatic amino acids in the lignification process, we analyzed the expression of several genes for lignin biosynthesis in wheat samples in the presence of PAL substrates as well as potential inhibitors (Figure 5). The expression of the *PAL6*, *C4H1*, *4CL1*, and *C3H1* genes significantly correlated with the lignin content in wheat samples (Figure 6).

Plants exposed to nutrient media additionally enriched with PAL substrates (L-Phe and L-Tyr) showed a significant increase in the number of transcripts of almost all genes selected for analysis.

However, the level of *C4H1* gene transcripts, the protein product of which is responsible for the conversion of trans-cinnamic acid (TCA) to p-coumaric acid (PCA), was an exception. In plants exposed to the medium supplemented with L-Tyr, the relative expression of the *C4H1* gene was comparable to the level of expression of this gene in plants of the control group. In contrast, the level of *C4H1* gene expression in plants incubated on a medium additionally enriched with L-Phe was significantly higher than the control by about 2 times. This is probably due to the fact that the transformation of L-Tyr influenced by PAL leads to the direct formation of PCA.

The expression level of the *PAL6* gene in plants grown in the presence of the main substrate’s stereoisomer (D-Phe) was significantly higher than in the control. However, the expression of all other genes selected for analysis was comparable to the expression level of plants from the control group.

Plants incubated on the OBHA-enriched medium show a reduced level of expression of genes below *PAL6* in the metabolic pathway compared to the control. OBHA, acting as a competitive inhibitor with respect to PAL, most likely limits the amount of substrate for subsequent transformations by controlling the expression of lignin-associated genes through a feedback loop.

The presence of key amino acid substrates (L-Phe and L-Tyr) in the nutrient medium led to a significant increase in the number of *PAL6* gene transcripts. This gene encodes the synthesis of an enzyme that potentially catalyzes the conversion of both amino acids. Thus, it can play a key role in the subsequent regulation of the synthesis of monolignol structures. This is confirmed by the increased expression of genes located downstream of the metabolic pathway. Contrastingly, OBHA and D-Phe either stimulated the synthesis of transcripts of individual genes or their presence did not lead to an increase in the number of transcripts of genes associated with lignin synthesis at all.

## 3. Discussion

### 3.1. Rationale for the Choice of the Ontogenesis Stage

Phases 4 and 5 on the Feekes scale of the cereal ontogenesis are characterized by the onset of erect growth, the establishment of stem structures, and, as a consequence, the establishment of a secondary cell wall, which prevents the lodging of cereals [23]. As is well known, the secondary cell wall is characterized by the presence of lignin—a phenolic biopolymer that determines multiple physiological properties at this and subsequent stages [24]. In addition, these stages are characterized by the vulnerability of plants to infectious agents and phytophages [23]. Thus, the establishment and intensification of lignin synthesis, as a nonspecific factor of immunity, also plays a fundamental role at this stage of plant development [25]. It was shown that mutants of Arabidopsis with knockouts in genes associated with lignin synthesis were characterized by a decrease in basal resistance, or effector-induced resistance, to microbial pathogens [26,27].

### 3.2. Rationale for the Choice of Genes for Analysis

Phe and Tyr are the shikimate pathway products of higher plants. The first mandatory step towards the formation of phenylpropanoids is the deamination of Phe into cinnamate by PAL. The subsequent formation of coumarate involves the direct hydroxylation of the aromatic ring by trans-cinnamate 4-hydroxylase (C4H). It should be noted that, when influenced by the bifunctional PAL (PTAL) of cereals, Tyr can be directly converted into coumaric acid, bypassing the stage of cinnamate formation. This matches the results showing that the amount of C4H transcripts was reduced in plants cultivated on Tyr-enriched media. However, the p-coumarate (obtained as a result of one or two actions) acts as a substrate for enzymes downstream of the metabolic pathway [16].

That way, p-coumarate-3-hydroxylase (C3H) converts p-coumaroyl shikimate to caffeoyl shikimate [28]. As 4-coumarate-CoA ligase (4CL) is one of the key intermediates in the biosynthesis of monolignols, it catalyzes the formation of activated thioesters of hydroxycinnamic acids [29]. This is reflected in the obtained results showing that the expression of the *C3H1* and *4CL1* genes significantly increases, in comparison with the control, in plants incubated on nutrient media containing L-Phe and L-Tyr. On the contrary, the number of transcripts of lignin-associated genes decreased in the presence of the hydroxylamine equivalent of phenylalanine (which acts as a PAL inhibitor).

### 3.3. The Role of PAL in the Formation of Lignin Structures

PAL is one of the important enzymes that catalyze the transformation of substrates to cinnamic acid (or p-coumaric acid) at the initial stage of the biosynthesis of lignin precursors. It was shown that a decrease in the PAL and C4H activity in tobacco (*Nicotiana tabacum*) via antisense suppression led to both a decrease in the content and a change in the subunit composition of lignin [30]. The use of 2-aminoindan-2-phosphonic acid (AIP) and L-α-aminooxy-β-phenylpropionic acid (AOPP)—the PAL-selective inhibitors—at low concentrations (100 μM) inhibited lignin accumulation without interfering with the differentiation of tracheary elements. Moreover, these inhibitor concentrations did not have any effect on the accumulation of cellulose and hemicellulose [31].

This is also indicated by the research on obtaining plant mutants in the key genes for the monolignol synthesis, PAL in particular. Such mutations result in defective phenotypes such as dwarfism and male sterility, which means lignin is critical for plant growth and development [32,33]. Multiple studies show that the downregulation of *PAL*, *C4H*, *4CL*, and *C3H* has a noticeable effect on the lignin content [34,35,36].

Lignin deficiency can lead to a variety of physiological changes. Lignin is an important part of the formation of the vascular system in plants, and, notably, it ensures adequate water transport. The cross-linking of cell wall polysaccharides with lignin, which is more hydrophobic, reduces the uptake of water by the cell wall and allows the vascular tissues of plants to run water efficiently [37]. Cross-linking enhances structural support of the whole plant as well as resistance to cell collapse under the tension of water transport [38].

Our results also demonstrate a direct relationship between PAL activity, the number of *PAL6* transcripts, and the level of lignin in the cell wall of wheat plants at the early stage of ontogenesis.

### 3.4. Assessment of the Role of POD and Hydrogen Peroxide in the Lignification Process

After primary biosynthesis, monolignols polymerize to form a lignin polymer. It is assumed that peroxidases play an important enzymatic role in catalyzing the polymerization of monolignol [39]. Our research has shown an increase in peroxidase activity in wheat plants that intensively accumulate lignin in the tissues.

There are a number of studies demonstrating increased expression of peroxidase genes in tissues undergoing intense lignification [40]. Mader and Anberg-Fisher showed that peroxidases have the ability to polymerize cinnamyl alcohols in the presence of hydrogen peroxide [41]. However, our results do not demonstrate an increase in the level of hydrogen peroxide in plants exposed to aromatic amino acids. On the contrary, the highest concentration of hydrogen peroxide was observed in plants with a reduced ability to form lignin (exposed to OBHA).

The role of peroxidases in the process of lignification is confirmed, among other things, by studies on transgenic plants with altered peroxidase activity. For instance, it was shown that the expression of the tomato peroxidase gene in transgenic tobacco led to an increase in the lignin content [42]. Moreover, transgenic plants with overexpressed peroxidase showed a higher level of lignin compared to wild-type plants [43]. On the other hand, mutants with a single or double knockout in the peroxidase genes show a reduced lignin content and/or a qualitative change in the lignin composition in Arabidopsis plants [44]. So, for example, the *prx72* mutant demonstrates a decrease in the lignin content by ~35%, compared to the plants of the control group [45].

### 3.5. Assessment of the Role of Inhibitors in the Lignification Process

There are a few selective inhibitors of the phenylpropanoid pathway enzymes: 2-aminoindan-2-phosphonic acid (AIP), (l-amino-2-phenylethyl)phosphonic acid (APEP), L-α-aminooxy-β-phenylpropionic acid (AOPP, a competitive inhibitor of the PAL enzyme), piperonylic acid (PIP, a quasi-irreversible inhibitor of the C4H enzyme) and 3,4-methylenedioxycinnamic acid (MDCA, a competitive inhibitor of the 4CL enzyme) [46,47].

In our study, the optical isomer of L-Phe—D-Phe—and the hydroxylamine equivalent of phenylalanine—O-Benzylhydroxylamine (OBHA)—were used as inhibitors. The data allow us to make a reasonable conclusion, albeit based on a set of indirect evidence, that D-Phe does not interfere with the synthesis of lignin at such a concentration. The presence of D-Phe in the nutrient medium led to an increase in PAL activity (0.58 µg TCA mg proteins^−1^ h^−1^ in comparison with the level of control plants: 0.38 µg TCA mg proteins^−1^ h^−1^), an increase in the level of cell wall lignification in experimental plants and an increase in the level of the expression of the *PAL6* gene. However, the level of transcripts of genes downstream of the metabolic pathway is not affected as much by this compound. As shown in the work of Szkutnicka and Lewak, the addition of D-Phe to the culture medium stimulated the PAL activity and prevented the decrease in the enzyme activity in the future [48]. It is possible that such a stimulating effect of the stereoisomer of the main PAL substrate is not directly related to either germination or the growth of wheat seedlings. The increase in PAL activity observed in the presence of D-Phe is likely to occur by a similar mechanism in different plants. The products of the PAL catalyzed reaction induce the synthesis of the PAL inactivating system. We can hypothesize that D-Phe, a competitive inhibitor of PAL, prevents the accumulation of these products and thus inhibits the PAL inhibitor complex formation. However, at the same time, D-Phe also stimulates the expression of lignin-associated genes. So, in these conditions, the stimulation of PAL by typical substrates leads to the intensification of lignin accumulation.

It was shown, using the model plant of *Brachypodium*, that coumarate (the end product of the tyrosine ammonia-lyase reaction) can inhibit both the PAL and TAL activity of bifunctional PTAL, but it does not affect the activity of monofunctional PAL [15]. This may indicate that PAL and TAL functions in cereals may be regulated differently by certain lignin intermediates.

As shown in in vivo experiments, OBHA significantly inhibits PAL activity. Hoagland’s experiments demonstrated that this inhibitor effectively reduces the activity of PAL (obtained by extraction), whereas its effect on plant growth was insignificant [19]. Previously, OBHA was studied in plant systems only as an ethylene inhibitor. Since PAL activity is altered by ethylene, research on the correlation between ethylene (altered by OBHA) levels and PAL activity may be quite promising [49]. Evidently, OBHA led to a significant decrease in the lignin level of experimental plants as the enzymatic activity of PAL was suppressed.

## 4. Materials and Methods

### 4.1. Triticum aestivum L. Cultivation and Experimental Design

It is a known fact that the PAL enzyme of angiosperms uses L-Phe as the main substrate; however, tyrosine ammonia-lyase activity has been discovered in a number of monocotyledonous plants. PAL can deaminate L-Tyr and use this amino acid as an alternative substrate in lignin biosynthesis [50].

The use of inhibitors in physiological and metabolic studies is fairly common [18]. As potential PAL inhibitors, several specific compounds were selected, for example, hydroxylamine equivalents of phenylalanine—O-Benzylhydroxylamine and D-phenylalanine—the optical isomer of L-phenylalanine [19,20].

The wheat (*Triticum aestivum* L.) “Vánek” variety was obtained from the seed station at the Ministry of Agriculture of the Kaliningrad region. Conditioned seeds were selected for the experiment, without visible mechanical damage and/or damage of another nature. The seeds were sterilized by incubating for 2 h in 10% (*v/v*) NaClO, then washed at least 10 times with distilled water.

In order to study the separate effect of the PAL substrates and inhibitors on the metabolic processes of *Triticum aestivum* plants, throughout the experiment, they were incubated on nutrient media containing 500 μM of the corresponding compounds (Figure 5).

The choice of the concentration of active substances is not accidental; it is based on research done by the team earlier [17]. Around 3–4 days after sprouting, the seedlings were transplanted onto a liquid, intensively aerated nutrient medium (2 L pots, with 4–5 plants each) containing 50% Hoagland’s solution, with the addition of the corresponding compounds. Samples incubated on the nutrient media without any additives acted as a control group. The nutrient solutions were refreshed twice a week. The plants were grown in the climatic chamber (Binder KBWF 720, GmbH, Tuttlingen, Germany) under fluorescent lamps at a photon flux density of 200 μmol m^−2^ s^−2^, for 16 h photoperiod and a day/night temperature of 25 °C.

All the samples were analyzed when the plants reached the age of 24 days (which corresponds to Phases 4 and 5 on the Feekes Scale). Vegetative photosynthetic parts of plants were used for the experiments. The tissues were frozen in liquid nitrogen immediately after harvest and then stored at −80 °C until further use.

### 4.2. PAL/TAL Activity Assays

Protein extraction and kinetic tests were performed as described by Cheng and Breen [51]. Plant material weighing 0.2 g was flash-frozen in liquid nitrogen and stored at −80 °C. Then, the sample was ground and washed with 1 mL of ice-cold acetone. The resulting mixture was incubated at −20 °C for 15 min and centrifuged at 16,000× *g* for 15 min at 4 °C. The precipitate was obtained by gentle rotation at 4 °C in the presence of 100 mM borate buffer. After 1 h, the samples were centrifuged again as described above, and the supernatant was used as the plant extract in kinetic analyses.

PAL activity was quantified by the production of trans-cinnamic acid (TCA), which was controlled by collecting the absorption data at 290 nm [52] every minute for 20 min at 37 °C using UV-3600, Shimadzu, Japan. The mixture contained 61 mM L-phenylalanine, 30 mM sodium borate buffer (pH 8.8), and 75 μL of plant extract, making the total volume of the resultant mixture 1 mL. The substrate was added after a 10 min pre-incubation period at 37 °C. The plant extract, pre-incubated in buffer without substrates, was used as a blank solution prior to each assay. Each sample was analyzed in duplicate.

TAL activity was determined in a similar way, by monitoring the production of p-coumaric acid (PCA) at 310 nm [52]. The 1 mL assay mixture contained 1.9 mM L-tyrosine, 30 mM sodium borate buffer (pH 8.8) and 75 μL of the plant extract. The temperature and blank solution preparation were identical to the PAL analysis.

The enzyme activity was expressed in units per mg of protein. One unit of PAL activity was defined as the amount of enzyme that produced 1 μg of cinnamic acid per hour. One unit of TAL activity was defined as the amount of enzyme that produced 1 μg coumaric acid per hour. Substrates—l-phenylalanine and l-tyrosine—and products—trans-cinnamic acid (TCA) and p-coumaric acid (PCA)—were used as standards, respectively.

### 4.3. Peroxidase Activity Assay

The peroxidase activity assay was performed using the Malik and Sing method with some modifications [53]. First, 100 μL of extract and 1 mL of *o*-dianisidine solution were added to 2 mL of phosphate buffer (pH 6.0/pH 7.0). The reaction was started by adding 100 μL of 0.2 mM hydrogen peroxide (H_2_O_2_), and, subsequently, the absorbance was measured at 460 nm every 30 s for up to 5 min. The enzyme activity was performed using an extinction coefficient of *o*-dianisidine. The POD activity was expressed as unit per mg of protein. Protein concentrations for both tests were determined by the Bradford method [54].

### 4.4. Hydrogen Peroxide Concentrations Assay

The concentration of hydrogen peroxide in plant samples was determined using the methods described by S. Sharma et al. [55]. Plant tissues were homogenized in the presence of trichloroacetic acid in an ice bath. Then, the homogenate was centrifuged at 16,000× *g* for 15 min. The resulting supernatant was used to determine the concentration of H_2_O_2_. The assay mixture contained the supernatant, 10 mM phosphate buffer (pH 7.0), and 1 M potassium iodide (KI). The resulting mixture was incubated in the dark for 1 h, then absorbance was measured at 390 nm. The H_2_O_2_ concentration was determined using a standard calibration curve and expressed as μg H_2_O_2_ g^−1^ of fresh weight.

### 4.5. Lignin Content

Evaluation of the lignin content was carried out after appropriate preparation of the sample tissues. It is necessary to remove all UV-absorbing compounds (primarily proteins) to avoid interference with the absorption maximum for lignin at 280 nm. Dry samples (0.3 g) were homogenized in the presence of 50 mM potassium phosphate buffer and then centrifuged. Then, the precipitate was washed several times and sequentially centrifuged after each washing procedure: twice in the presence of phosphate buffer (pH 7.0), three times in the presence of 1% Triton X-100 detergent, twice with 1 M NaCl buffer at pH 7.0, twice with distilled water and twice with acetone. The precipitate was dried in a drying oven (60 °C, 24 h) and cooled in a vacuum desiccator. The resulting dry matter was determined as the fraction of the cell wall that does not contain proteins [56].

A sample of the cell wall (20 mg) was placed in a centrifuge tube with 0.5 mL of 25% acetyl bromide (*v/v* in glacial acetic acid), then incubated at 70 °C for 30 min. After complete decomposition, the sample was rapidly cooled in an ice bath and then mixed with 0.9 mL of 2 M NaOH, 0.1 mL of 5 M hydroxylamine-HCl and glacial acetic acid, sufficient to completely solubilize the lignin extract. After centrifugation (1400× *g*, 5 min), the absorbance of the supernatant was measured at 280 nm [57]. The results were expressed in mg of lignin g^−1^ of the cell wall.

### 4.6. Assessment of the Level of Expression of Lignin-Associated Genes

Total RNA was extracted from plant samples using Trizol (Invitrogen, Carlsbad, CA, USA).

For cDNA synthesis, RNA samples were treated with DNase 1 (Thermo scientific, Waltham, MA, USA). The 10 μL reaction mixture containing 1 μg of RNA, 1 μL 10× of reaction buffer and 1 e.a. of DNase was incubated at 37 °C for 15 min. DNase was inactivated by adding 2.5 mM EDTA and heating at 65 °C for 10 min. Single-stranded cDNA was synthesized from total RNA using the Revert Aid H minus First Strand cDNA Synthesis Kit (Thermo Scientific, USA) as described by the manufacturer [58]. The cDNA concentration was assessed spectrophotometrically.

Real-time polymerase chain reaction (PCR) was performed on a CFX96 ™ Real-Time System (Bio-Rad Laboratories, Hercules, CA, USA) using the SYBRGreen I intercalating dye (Invitrogen, USA). Primer sets were designed from wheat sequences deposited in GenBank [59]. The ΔΔCt method was used to estimate the relative expression levels of the analyzed genes [60].

Previously, it was shown that the expression of *PAL6*, *C4H1*, *4CL1*, and *C3H1* correlates with the level of lignin in wheat plants [61]. *ARF* (ADP-ribosylation factor) and actin were chosen as comparison genes. A list of primers for selected genes is provided in Supplementary Table S1.

### 4.7. Statistical Analysis

The values are means of at least three replicates. The level of significance was established at a *p*-value of *p* ≤ 0.05. Data were statistically analyzed by using the SigmaPlot 12.3 (Systat Software GmbH, Erkrath, Germany). The Shapiro–Wilk test was used for normality checking; additionally, the experimental data were checked for the homogeneity of variance.

### 4.8. Reagents

All analytical-grade chemicals used in the assay were obtained from commercial sources. Bi-distilled water was used throughout the experiment. Standard solutions and nutrient media were prepared by dilution of the stock solution.

## 5. Conclusions

This study analyzed the participation of PAL in the formation of lignin at the early stages of ontogenesis, prior to the establishment of stem structures. It is safe to say that selective stimulation of PAL by its substrates led to an intensification of lignin accumulation, as well as an increase in the differential expression of lignin-associated genes. The substances selected as potential inhibitors had contradictory effects. One of them, D-Phe, exhibited physiological effects similar to those of characteristic PAL substrates. Not only did it not cause a decrease in lignin production, but, on the contrary, it stimulated its formation. In addition, D-Phe stimulates the expression of lignin-associated genes. Although several studies indicate the inhibitory properties of this compound, it is clear that D-Phe is not suited for this role in wheat studies. However, the very mechanism of D-Phe stimulating secondary metabolism is interesting and deserves additional attention. On the other hand, OBHA effectively blocks PAL activity and significantly influences downstream reactions. For example, the level of lignin in the cell wall decreased dramatically. This effect emphasizes the exceptional role of PAL in the synthesis of phenylpropanoids in general, and lignin in particular.

In addition to the physiological functions of lignin, it should be noted that it is also the main component of plant biomass. Today, it is regarded as a promising source of renewable aromatic precursors for chemical technology. Thus, the search for ways to intensify the biosynthesis of this aromatic polymer remains an urgent task of modern biotechnology.

## Figures and Tables

**Table 1:** Effect of different substances (substrates and potential inhibitors) on growth processes of wheat plants.

	Seed Germination, %	Plant Height, cm	Shoot Fresh Weight, G	Shoot Dry Weight, G	Dry Matter Content, %
Control	56 ± 9.4 ^a^	18.4 ± 1.2 ^c^	3.7 ± 0.4 ^c^	1 ± 0.1 ^c^	27%
L-Tyr	62 ± 6.5 ^a^	24.4 ± 2.4 ^ab^	5.5 ± 0.3 ^a^	1.7 ± 0.2 ^a^	31%
L-Phe	58 ± 8 ^a^	26.4 ± 2.2 ^a^	5.8 ± 0.3 ^a^	1.8 ± 0.2 ^a^	31%
OBHA	68 ± 7.5 ^a^	13.1 ± 3.4 ^d^	2.1 ± 0.2 ^d^	0.4 ± 0.1 ^d^	19%
D-Phe	66 ± 9 ^a^	22.6 ± 1.4 ^b^	4.7 ± 0.3 ^b^	1.4 ± 0.2 ^b^	30%

Values marked with different letters show significant differences at (*p* ≤ 0.05) according to Tukey’s test.

## Supplementary Materials

The following are available online at https://www.mdpi.com/article/10.3390/ijms22189848/s1. Table S1: A list of primers for lignin associated genes.

## Abbreviations

4CL	4-coumarate-CoA ligase
AIP	2-aminoindan-2-phosphonic acid (AIP)
AOPP	L-α-aminooxy-β-phenylpropionic acid
APEP	(l-amino-2-phenylethyl)phosphonic acid
ARF	ADP-ribosylation factor
C3H	p-coumaroyl shikimate/quinate hydroxylase
C4H	cinnamate 4-hydroxylase
D-Phe	D-phenylalanine
L-Phe	L-phenylalanine
L-Tyr	L-tyrosine
MDCA	3,4-methylenedioxycinnamic acid
OBHA	O-Benzylhydroxylamine
PAL	phenylalanine ammonia-lyase
PCA	p-coumaric acid
PIP	piperonylic acid
POD	peroxidase
qRT-PCR	quantitative real-time PCR
SAR	systemic acquired resistance
TAL	tyrosine ammonia-lyase
TCA	trans-cinnamic acid
