# Supplementary material for: Assessment of the Role of PAL in Lignin Accumulation in Wheat (Tríticum aestívum L.) at the Early Stage of Ontogenesis
Source: Int J Mol Sci. 2021 Sep 12;22(18):9848. doi: 10.3390/ijms22189848 (PMC8470810; doi:10.3390/ijms22189848)
Supplement: Supplementary file 1 [file ijms-22-09848-s001.zip › ijms-1358931-supplementary.pdf]

Supplementary Material

Table S1: A list of primers for lignin associated genes

**Lignin related genes**		
	Primer	Sequence (5′ -3′)
**PAL6**	PAL6-F	CTCAAGCTCATGTCCTCCACA
	PAL6-R	TCAGCACCTTCTTCGACACC
**C3H1**	C3H1-F	GGCTGTGTCCACTTAATG
	C3H1-R	TGTCATCACTAAGGTCATAC
**C4H1**	C4H1-F	CAGCCTCCACATCCTCAAG
	C4H1-R	CTTAGGACGAGCGAACAATC
**4CL1**	4CL1-F	CACTCAGCCAGCCAGCAG
	4CL1-R	ACATTACACAAGCAGGAAGAACC
**Reference genes**		
**Actin**	ACT-F	GCCACACTGTTCCAATCTATGA
	ACT-R	TGATGGAATTGTATGTCGCTTC
**ARF**	ARF-F	CTGACGCCGAGGATATCCA
	ARF-R	GCCTTGACCATACCAGTTCCA
